# Dignity and support after cancer: we need to talk about palliative care

**DOI:** 10.1016/j.lana.2024.100719

**Published:** 2024-03-14

**Authors:** The Lancet Regional Health – Americas

Feb 4 was World Cancer Day, and although this should be a day to focus on inspiring change and action, just-released estimates for new cancer cases are worrisome, with 35 million new cancer cases predicted worldwide for 2050. A 142% increase in cancer incidence is expected in low Human Development Index countries and a 99% increase is expected in medium Human Development Index countries, highlighting inequalities between these countries. Cancer is one of the main causes of severe health issues in Latin America and the Caribbean (LAC), with more than 3.5 million people needing palliative care (PC) per year. Despite being implemented in many countries, there is a clear gap in equitable access, with many barriers to accessing PC and end-of-life support in LAC.

According to the Pan American Health Organization, PC is an approach to deal with chronic conditions and end-of-life care by managing pain and other physical symptoms and assisting patients and families with psychosocial and spiritual support. However, access to PC is still an ongoing problem in LAC countries, with only 7% of patients receiving this kind of support. Despite being mostly focused on cancer patients, PC is recognised as a public health concern that includes many other life-threatening diseases. To strengthen health systems and improve quality of life, PC needs to be fully integrated into health systems.

The integration of PC in LAC has been inconsistent, with difficult access for patients. In 1990, WHO released a model addressing four components to integrate PC in existing health-care systems: (i) appropriate policies, (ii) adequate drug availability, (iii) education of health-care workers and the public, and (iv) implementation of PC services at all levels of society. PC has grown in Brazil, the largest country in the region, over the past 15 years and, on Dec 7, 2023, a resolution was approved to implement the National Policy of Palliative Care in the public health system (Sistema Único de Saúde), making it freely available to all. However, most of the services in the country are still concentrated in hospitals, leaving a need to reach to other departments, especially in primary care. Regarding education, only eight countries from the region include PC as a specialisation incorporated in graduate and postgraduate programmes, highlighting the lack of preparedness and access to education for medical practitioners.

According to the Atlas of Palliative Care, there are 922 PC services, units, or teams in LAC (1.63 per one million inhabitants), with the most services in Chile, which ranks as one of the countries with the best end-of-life care and access to PC in LAC. Despite the advances in implementing PC in LAC, several challenges still exist with most of the population experiencing heterogeneity in terms of availability and access to PC. In Chile, for example, insurance for PC is restricted to patients with cancer, those who are terminally ill, and patients with cancer that are suffering from pain, with a mix of access from public and private insurances. This prevents the population from having total access to PC and achieving universal health care.

From the patient’s perspective, barriers such as cultural stereotypes, lack of education, and stigma surrounding disease can discourage them from seeking proper treatment and care. One way to close the gap between patients and treatment is by promoting and implementing a multidisciplinary approach and ultimately calling for a restructure of health systems. This could be achieved by increasing financial coverage and organisational restructuring, which could be strategically effective with implementation of PC in primary care. Implementation should not be done only by specialists but also by general practitioners, as it pertains to different areas inside medical practice. Actions and recommendations to overcome the barriers that were highlighted in a *Lancet Oncology* commission in 2015 were proposed and discussed by *The Lancet* series on Cancer Control in LAC. These actions included implementation of PC legislation, access to opioids, and courses focusing on PC in medical schools. Some improvements were seen in the region after the commission, including an increase in service availability from 1.6 to 2.6 per million inhabitants, an increase in national legislation from seven to ten countries, and better access to pain medication. However, other challenges still exist to meet the needs of patients, such as availability of ambulatory PC programmes and appropriate access to drugs throughout the region.

Examples of approaches to minimise the burden and provide adequate support for patients that require PC intervention are strengthening health-care systems and improving the infrastructure and availability of services. PC is a core component of universal health care and a human right that should cover all kinds of diseases, and paucity of access to such care restricts people from having adequate support during life-threating conditions and even at end of life. With the predicted increase of cancer cases, the number of patients that need PC will rise, and if LAC is not prepared, those patients will suffer due to the paucity of adequate care. The extent of the burden can be lessened if governments prioritise community development and engagement, include PC as a multidisciplinary intervention that should cover different areas of medical practice (from generalist to specialist), and ultimately focus on a patient and family-centred approach by providing not only treatment but also psychosocial and spiritual care, increasing the patient–doctor–family connection. Patients have the right to quality of life, to have their pain and suffering minimised, and ultimately, to have a voice during their care and a dignified end-of-life.

